# The experiences of, and need for, palliative care for people with motor neurone disease and their informal caregivers: A qualitative systematic review

**DOI:** 10.1177/0269216320908775

**Published:** 2020-04-14

**Authors:** Kate Flemming, Victoria Turner, Samantha Bolsher, Bill Hulme, Elizabeth McHugh, Ian Watt

**Affiliations:** 1 Department of Health Sciences, Faculty of Science, University of York, York, UK; 2 Expert by Experience, York, UK; 3 St Leonard’s Hospice, York, UK

**Keywords:** Motor neurone disease, amyotrophic lateral sclerosis, qualitative research, systematic review, palliative care, caregivers

## Abstract

**Background:**

Despite being a terminal neurodegenerative disease, the role of palliative care is less recognised for motor neurone disease than for other life-limiting conditions. Understanding the experiences of, and need for, palliative care for patients and carers is key to configuring optimal policy and healthcare services.

**Aim:**

To explore the experiences of, and need for, palliative care of people with motor neurone disease and their informal carers across the disease trajectory.

**Design:**

A systematic review of qualitative research conducted using Thematic Synthesis – PROSPERO registration CRD42017075311.

**Data Sources:**

Four electronic databases were searched (MEDLINE, CINAHL, PsycINFO, Social Science Citation Index) using terms for motor neurone disease, amyotrophic lateral sclerosis, palliative care, and qualitative research, from inception to November 2018. Included papers were data extracted and assessed for quality.

**Results:**

A total of 41 papers were included, representing the experiences of 358 people with motor neurone disease and 369 caregivers. Analytical themes were developed detailing patients’ and carers’ experiences of living with motor neurone disease and of palliative care through its trajectory including response to diagnosis, maintaining control, decision-making during deterioration, engaging with professionals, planning for end-of-life care, bereavement.

**Conclusion:**

The review identified a considerable literature exploring the care needs of people with motor neurone disease and their carers; however, descriptions of palliative care were associated with the last days of life. Across the disease trajectory, clear points were identified where palliative care input could enhance patient and carer experience of the disease, particularly at times of significant physical change.

**What is already known about the topic?**

- Motor neurone disease is a life-limiting condition from the point of diagnosis.
- The role of palliative care is less recognised for motor neurone disease than for other life-limiting conditions.
- Patient and carer experiences and palliative care needs across the motor neurone disease trajectory have not been adequately explored.

**What this paper adds?**

- Patient and carer experiences of palliative care tend to occur at the ‘end-of-life’ stage of the motor neurone disease.
- Diagnosis was recalled as a very vulnerable time by both patients and carers where they would have valued additional support and the potential involvement of palliative care.
- Patients’ and carers’ described their experiences of motor neurone disease as a cycle of unremitting loss and uncertainty.

**Implications for practice, theory or policy**

- Palliative care support for people with motor neurone disease should be reframed to start at the point of diagnosis.
- Early and effective communication, with a single point of contact, can help anticipate and plan intervention options to manage deterioration and reduce uncertainty.
- Healthcare professionals require support and education to initiate advance care planning early in the disease and to provide support around decision-making at points of deterioration.

## Background

Motor neurone disease, or amyotrophic lateral sclerosis, is a neurodegenerative idiopathic disease with sudden onset and continual deterioration resulting in complex and disabling symptoms and care needs.^1^ It is currently without cure, with the average time between symptom onset and death being 2–4 years and the progression of symptoms and onset of disability is often measured over weeks and months rather than years.^2^ The terminal nature of the disease justifies initiating supportive palliative care early in the disease trajectory. Historically, however, while a palliative care approach has been advocated for care of people with life-limiting illnesses, such as cancer, it has been less recognised as an approach relevant for patients with motor neurone disease or other neurodegenerative disorders.^2^ This picture is now changing; the progressive and terminal nature of motor neurone disease, the need for anticipatory planning and regular re-evaluation of symptoms and the central role of carers are being seen as highly relevant to the specialism of palliative care.^2^ With this comes recognition that palliative care should be initiated early in the disease trajectory, rather than waiting until near the end of life, with the involvement of palliative care specialists to help with advance care planning and symptom control.^3–5^

Given the potential for rapid disease progression and deterioration associated with motor neurone disease, it is essential to have a clear understanding of individuals’ experiences of palliative care alongside the potential needs and priorities for such care across the disease trajectory. Existing research in this area includes an exploration of people’s experiences of living with motor neurone disease through a systematic review of qualitative research. The review of 20 qualitative studies identified how individuals become experts of their disease and seek to maintain control over it; however, it did not focus on either palliative care needs or the experiences of carers.^6^ A further review, in the form of guidance for the assessment and management of motor neurone disease from patients’ and carers’ perspectives, briefly details the preferences and concerns about care at the end of life at trigger points such as diagnosis, whether there is a significant change in physical function or whether interventions such as gastrostomy or non-invasive ventilation are needed.^5^ The targeted focus of this review meant that wider palliative care needs, such as symptom management and family support, were not addressed. However, unique challenges specific to distinct stages of the disease trajectory have been described^7,8^ and determining palliative care needs according to disease stage has rarely been explored in the literature.^9^

There is currently an identified gap in synthesised knowledge in this area.^10^ The methodologies associated with qualitative research enable individuals’ perceptions and experiences about their illness and their care needs to be established.^11^ Systematic reviews of qualitative research enable existing research to be collated, developing new insights and identifying research gaps. The approach is particularly relevant to palliative care, as synthesising qualitative research allows maximum value to be gained from primary studies that have overcome challenges in accessing and researching vulnerable groups.^12^ By exploring the self-reported palliative and supportive care needs and experiences of both patients and carers through a synthesis of existing qualitative research studies, a greater understanding will be developed of these needs, in turn guiding future policy and service development.

## Aim

To explore the experiences of, and need for, palliative care for adult patients with motor neurone disease and their informal carers across the disease trajectory, through a systematic review of qualitative research.

## Methods

This review protocol was registered with PROSPERO CRD42017075311 (https://www.crd.york.ac.uk/prospero/display_record.php?RecordID=75311). The review was conducted using Thematic Synthesis, an approach to synthesising qualitative research papers, favoured here for its ability to formalise the identification and development of themes arising from included studies.^13^ The methods of thematic synthesis guide the processes of synthesis of findings and are not associated with other aspects of the review process. To ensure transparency of reporting for all stages of the review process, the review was reported in accordance with Enhancing Transparency in Reporting the synthesis of Qualitative (ENTREQ) research guidelines.^14^

### Search strategy

Searches for published and unpublished research were developed iteratively with an information specialist. The search strategy (Table 1) was developed using terms for motor neurone disease, amyotrophic lateral sclerosis, palliative care, and carers and used a qualitative research filter.^15^ Searches were run in the electronic databases CINAHL, MEDLINE, PsycINFO, and Social Science Citation Index from inception until 8 June 2017, with an updated search undertaken in November 2018. We also used specific ‘ahead of print’ searches in PubMed and Google Scholar to ensure that the searches remained up to date throughout the project period. Electronic searches were supplemented with citation searching and consultation with the project team for known research. Screening was undertaken by one reviewer (V.T.) and a random selection of 30% of the results checked by a second reviewer (K.F.).

**Table 1.** Search strategy for MEDLINE.

Via OVID, search date 8 June 2017, records identified 256
Database: Ovid MEDLINE(R) Epub Ahead of Print, In-Process and Other Non-Indexed Citations, Ovid MEDLINE(R) Daily and Ovid MEDLINE(R) <1946 to Present>
1 exp Motor Neuron Disease/ (23,769)
2 Amyotrophic Lateral Sclerosis/ (15,876)
3 (motor adj2 neuron* adj2 disease).ti,ab. (4883)
4 (motorneuron* adj3 disease).ti,ab. (25)
5 MND.ti,ab. (1675)
6 Amyotrophic lateral sclerosis.ti,ab. (18,683)
7 ALS.ti,ab. (21,267)
8 1 or 2 or 3 or 4 or 5 or 6 or 7 (39,560)
9 palliative care/ or terminal care/ or hospice care/ or hospices/ or respite care/ (75,653)
10 ((palliat$ or terminal or hospice* or respite or end of life) adj3 (care or caring)).ti,ab. (33,524)
11 Bereavement/ (4944)
12 Grief/ (8463)
13 (bereave$ or grief or griev$).ti,ab. (12,013)
14 9 or 10 or 11 or 12 or 13 (99,456)
15 Qualitative Research/ (34,357)
16 qualitative.ti,ab. (173,469)
17 findings.ti,ab. (1,689,490)
18 interviews.ti,ab. (135,853)
19 15 or 16 or 17 or 18 (1,907,896)
20 8 and 14 and 19 (120)
21 Caregivers/ (28,997)
22 (caregiv$ or care giv$).ti,ab. (55,490)
23 carer$.ti,ab. (11,678)
24 informal care.ti,ab. (1371)
25 befriending.ti,ab. (149)
26 (caretak$ or care tak$ or caretaking).ti,ab. (4826)
27 (caretak$ or care tak$ or caretaking).ti,ab. (4826)
28 (child$ adj2 (care or cares or caring or support or supports or supporting)).ti,ab. (25,404)
29 ((son or sons or daughter$ or friend$ or partner$ or spous$) adj2 (care or cares or caring or support or supports or supporting)).ti,ab. (4890)
30 ((husband$ or wives or wife or spouse$ or grandparent$ or grandchild$ or neighbour$ or neighbor$ or relatives or relations or families or family or familial) adj2 (care or cares or caring or support or supports or supporting)).ti,ab. (20,475)
31 ((parent$ or mother$ or father$ or maternal or paternal or filial) adj2 (care or cares or caring or support or supports or supporting)).ti,ab. (16,091)
32 ((peer or peers) adj2 (care or cares or caring or support or supports or supporting)).ti,ab. (3686)
33 21 or 22 or 23 or 24 or 25 or 26 or 27 or 28 or 29 or 30 or 31 or 32 (135,623)
34 8 and 19 and 33 (200)
35 20 or 34 (256)

MND: motor neurone disease.

### Inclusion criteria

Studies were included in the review if they

- Used qualitative research methods to explore the experiences of, or need for, palliative care of adults with motor neurone disease and/or current or bereaved informal caregivers of adults with motor neurone disease across the disease trajectory.
- Were published in English.

Papers using both quantitative and qualitative methods of data collection were included if the qualitative data were reported separately and could be clearly extracted. There were no date limits set.

### Data extraction and quality appraisal

Relevant data were extracted from the included papers (aim, type and number of participants, methodology, methods of data collection, analysis and results) (Table 2). Data extraction was undertaken by one reviewer (V.T.) and checked by a second reviewer (K.F.). Papers were appraised for quality by two reviewers according to criteria by Hawker and colleagues^16^ with disagreements resolved by consensus. There was no a priori quality threshold for excluding papers given that studies of lower methodological quality can still contribute credible and transferable data.^17^ However, quality assessment was undertaken to ensure transparency in the process and to align with best practices (Table 2).^17^.

**Table 2.** Detail of included studies.

Study	Aim	Trajectory areas covered	Methodology	Data collection method	Participants (number, type)	Details of participants	Analysis	Results (themes)	Quality score
Galvin et al.^18^ Ireland	To explore the journey from first problem symptoms to diagnosis from the perspective of informal caregivers providing care to people with ALS	First symptoms, diagnosis	Thematic analysis	‘SSI’ – only single qualitative question asked	74 caregivers	Caregivers (*n* = 74) Age Mean: 55.7 (SD: 12.82) Range: 25–76 years Sex Male 23 (31.1%) Female 51 (68.9%) Relationship to the patient Spouse/partner 53 (71.6%) Son/daughter 15 (20.3%) Parent 2 (2.7%) Sibling 3 (4.1%) Friend 1 (1.4%) Lives with patient Yes 60 (81.1%) No 14 (18.9%) Principal economic status Working for payment or profit 32 (43.2%) Unemployed 4 (5.4%) Looking after family/home 16 (21.6%) Retired 19 (25.7%) Unable to work due to permanent sickness or disability 3 (4.1%) In general, would you say your health is Excellent 14 (18.9%) Very good 20 (27.0%) Good 26 (35.1%) Fair 9 (12.2%) Poor 5 (6.8%) Do you have any long-term illness, health problems or disability? Yes 39 (52.7%) No 35 (47.3%) *Patients* (*n* *=* *74*) *– N.B. not included in the study* *Age* *Mean: 65.16* (*SD: 9.74*) *Range: 43–87* *years* *Sex* *Male 45* (*60.8%*) *Female 29* (*39.2%*) *Site of onset*: *Bulbar 21* (*28.4%*) *Spinal 51* (*68.9%*) *Thoracic/respiratory 2* (*2.7%*) *Time from symptom onset to diagnosis* *Mean: (months) 15.72 (SD: 11.04)* *Range: 1–56* *months* *Median: 12* *IQR: 8–22*		Two main themes were identified: (1) problem signs and symptoms (A) noticing and (B) reaction; (2) interaction with the health services.	31
O’Brien et al.^19^ UK	To explore the personal perspectives of the diagnostic experience for people with ALS/MND and their family carers identifying issues that could impact positively or negatively on these experiences	Diagnosis	Thematic analysis	Narrative interviews	24 patients, 28 carers (18 current, 10 former)	Former carers: 6 female, 4 male Time since bereavement: 2 months to 7 years Current patients: 16 female, 9 male Age at diagnosis – 30–84 MND type – 3 PBP, 14 limb, 5 bulbar (−1 carer), 1 PMA, 1 PLS		Themes: symptom onset, experiences within primary care, diagnostic delays, communication of the diagnosis and responding to the diagnosis	33
Hugel et al.^20^ UK	To explore issues surrounding a new diagnosis for patients diagnosed with MND at a large regional neurosciences centre in the Northwest of England	Diagnosis		SSI	13 patients	9 male, 4 female Mean age: 64 (range: 33–79) 4 bulbar, 9 limb onset Median time from symptom onset to diagnosis = 8 months (range: 3–60 months)	Interpretive phenomenological analysis (IPA)	The major themes identified in descending order were ‘Family/carers’, ‘Communication of the diagnosis’, ‘Reaction to the diagnosis’, ‘Physical difficulties’, ‘Time before diagnosis’, ‘Information’, ‘Future’, ‘Coping with the diagnosis’ and ‘Formal support’.	33
O’Brien and Clark^21^ UK	To understand how personal spirituality and religious faith might help those living with amyotrophic lateral sclerosis/motor neurone disease (ALS/MND) cope with their impending death	Emotional adjustment	Thematic analysis	Unsolicited narratives (internet and print-published)	54 patients	Age at diagnosis: 21–77		Our findings reveal that religious faith sustains and helps people to avoid despair, and personal spirituality helps them make sense of what is happening to them.	30
Ozanne et al.^22^ Sweden	To illuminate experiences of finding meaning in life among spouses of people with amyotrophic lateral sclerosis	Emotional adjustment	Qualitative content analysis	SSI	13 spouses	Participating spouses were 38–87 years old (median: 68 years), and all lived with the ill person. The range of disease duration was 2–13 years		Main theme: struggling for meaning at the end of a dark tunnel Theme 1: feeling limited and isolated in the proximity of death (four subthemes) Theme 2: finding meaning despite the proximity of death (four subthemes)	30
Bolmsjo^23^ Sweden	To investigate the experiences of patients with ALS regarding their existential life situation.	Emotional adjustment	Hermeneutic	SSI	8 patients	6 female, 2 male Age range: 53–84		Five headings: experiences concerning meaning and guilt, experiences concerning relations, experiences concerning diagnosis and information, experiences concerning physical inability, experiences concerning dying with dignity and respect for the person	25
Foley et al.^24^ Ireland	To further our understanding of what loss means to people with ALS and how people with ALS exert control in their care in response to loss	Emotional adjustment		Qualitative interviews	34 patients	17 female, 17 male Age: 37–81 Duration since symptom onset 4 months to 13 years (average: 31 months) 27 lived with family, 7 alone or in care facility	Grounded theory	Headings: meaning of loss in ALS, exerting control over healthcare services	33
Locock and Brown^25^ UK	To explore attitudes to peer support among people with motor neurone disease (MND) and their family carers	Accessing services	Secondary analysis of two studies: Thematic analysis	Narrative interviewing with semi-structured prompting	48 patients, 22 carers	(data from original two papers) Brown study: Age range: 39–85 9 male, 4 female 2 PBP, 1 PMA, 2 PLS, 8 ALS Locock study: 35 people with MND, 11 family carers		Two overarching themes: valuing camaraderie and comparison, and choosing isolation	31
O’Brien et al.^26^ UK	Qualitative aim: to elucidate potential barriers to the uptake of such care (social services homecare) by analysing qualitative data from a larger sample of patients and carers than recruited by previous studies	Accessing services	(Mixed methods) Thematic analysis	Narrative interviews	24 patients, 28 carers (18 current, 10 former)	See O’Brien et al.^19^ (above)		Themes: entitlement, normality, care provision, understanding, putting off care, impact on carers	31
O’Brien et al.^27^ UK	To obtain the views of people with MND and family carers regarding multi-disciplinary team (MDT) working	Accessing services	Thematic analysis	Narrative interviews	24 patients, 18 family carers, 10 former carers	See O’Brien et al.^19^ (above)		Themes: having a single point of access, specialist knowledge and skills, saving time and energy, regular follow-up, valued members of the MDT, working together as a team.	26
Bakker et al.^28^ Netherlands	To explore the needs and value of case management according to patients with amyotrophic lateral sclerosis (ALS), their spousal caregivers and healthcare professionals in the context of multi-disciplinary ALS care	Accessing services	Thematic analysis	SSI	SSI: 10 patients, 10 carers, 10 HCPs Focus group (6 patients, 4 carers, 10 HCPs)	Patients 3 female, 7 male Age range: 51–74 years (mean age: 61 years) Disease duration: 0.6–66 months (mean: 28.5 months) All were married and had children		Two main themes: ‘needs for and receptiveness to case management’ and ‘appreciated aspects of case management’	33
Foley et al.^29^ Ireland	To identify key psychosocial processes that underpin how people with motor neurone disease engage with healthcare services.	Accessing services		Qualitative interviews	34 patients	Age: 37–81 17 female, 17 male Disease duration: ranging 4–169 months (average 31 months) Geographical location: rural (*n* = 19), semi-urban (*n* = 5) and urban (*n* = 10) Onset type: spinal (26), bulbar (6), respiratory (2)	Grounded theory	Key categories (control, reassurance, resignation and trust)	36
O’Brien and Preston^30^ UK	Little is known regarding the inpatient care received by patients. Our objective was to address this by exploring the experience of hospitalisation following a diagnosis of motor neurone disease from the perspective of family carers of those diagnosed with the illness	Accessing services	Secondary analysis of two qualitative studies (based on Locock and Brown)	Original studies used narrative-style interviews	10 bereaved carers (Study 1) 10 bereaved carers (Study 2*) 3 current carers (Study 1) *N.B. 2 of these are same as 2 bereaved carers from Study 1	Bereaved carers Study 1: 7 female, 3 male Time from bereavement – 2 months to 7 years Bereaved carers Study 2: 6 male, 3 female Time from bereavement – 5 months to 3 years 3 months Current carers from Study 1: 2 male, 1 female Disease duration 8 months to 30 months		Headings: lack of knowledge, basic care, reluctance for admission, final memories	32
O’Brien^31^ UK	To investigate the views of people at different stages in the progression of MND concerning their desire for information about their illness and to identify whether there was any pattern to information seeking among people with MND	Decision-making/information seeking		SSI	7 patients	4 female, 3 male Age range: 57–75 (mean: 66) 4 limb onset, 3 bulbar Time from diagnosis to interview 3–50 months (mean: 17 months)	IPA	Three types of information seeker: active seekers, selective seekers, information avoiders. Media coverage and unscreened information.	33
Burchardi et al.^32^ Germany	To explore how discussions about living wills are undertaken	Decision-making/information seeking		SSI	15 HCPs, 15 patients	Age (in years) Average 59 (43–78) Sex *n* (%) Male 10 67 Female 5 33 Marital status *n* (%) Single 1 (7) Married 13 (86) Widowed 1 (7) Education (German) *n* (%) High school (Hauptschule/Realschule) 11 (73) College (Abitur) 3 (20) Unknown 1 (7) Working profile *n* (%) Still working 2 (13) Incapable of working 10 (67) Retired 3 (20)	Grounded theory	Headings: Physicians’ and patients’ view of living wills, physicians’ and patients’ assessment of living wills, conditions for information and discussions about living Wills, appropriate time to discuss end-of-life care and living wills	33
Hagena et al.^33^ UK	To identify what information and support MND patients and their carers want and to determine whether there were barriers to taking part in	Accessing services, decision-making/information seeking	Not reported	Phase 1: focus groups Phase 2: postal questionnaires Phase 3: focused interviews	Phase 1: Phase 2: 18 patients Phase 3: 6 patients, 4 carers	Phase 1: 5 patients (4 female/1 male, age range: 59–84, time since diagnosis 1–6 years) 5 carers (3 female/2 male, 3 partners/2 children) 3 bereaved carers (2 F/1 M, all partners) Phase 3: 6 patients (5 F/1 M, age range: 46–78, time since diagnosis 8 months to 7 years) 4 carers (1 F/3M, all partners)	Not reported	Needs: information needs immediately after diagnosis, ongoing support and information needs, psychosocial support needs, barriers to taking part in support programmes	24
Hogden et al.^34^ Australia	To explore carer participation in decision-making, to identify carer roles, and determine the facilitators and barriers to carer participation in decision-making for ALS multi-disciplinary care	Decision-making/information seeking	Thematic analysis	SSI	8 carers	Relationship to patient Spouse = 5 Child = 2 Parent = 1 Duration of care (months) Range: 6–96 Mean: 40 Age (years) Range: 33–76 Mean: 56 Gender Male = 3 Female = 5 Employment status Working full-time = 4 Working part-time = 1 Not working/retired = 3		Three themes: Carer’s roles, facilitators of carer participation in decision-making and barriers to carer participation in decision-making	35
Hogden et al.^35^ Australia	To explore patient experiences of ALS and to identify factors influencing their decision-making in the specialised multi-disciplinary care of ALS	Decision-making/information seeking	Thematic analysis	SSI	14 patients	Age at interview (years) Mean: 60.5 (range: 40–77) Time from diagnosis to interview (months) Mean: 32.07 (range: 2–93) Gender Male 7 (50%) Female 7 (50%)		Decision-making was influenced by three levels of factors, i.e., structural, interactional, and personal.	35
Stavroulakis et al.^36^ UK	To explore the decision-making process leading up to gastrostomy insertion from the perspective of the patients and their informal carers	Decision-making/information seeking, Starting specific treatments	Retrospective qualitative exploration	SSI	10 patients, 8 carers	Patients: 3 male, 7 female Mean age: 67.1 (range: 42–91) 7 bulbar, 3 limb onset Mean duration of disease from onset to time of gastrostomy 26.5 months (range: 13.2–60.9)		Four headings: Factors triggering gastronomy decision (prolonged, tiring and effortful meals; the task of food preparation; choking and aspiration; weight loss) Factors delaying gastronomy decision (reluctance to give up oral feeding, uncertainty over the disease trajectory, not realising the potential benefits, negative perceptions of gastrostomy). Reflections on timing. Information to support decision-making.	34
Lemoignan and Ells^37^ Canada	To better understand the experience of decision-making about assisted ventilation for ALS patients	Decision-making/information seeking, starting specific treatments	Phenomenological approach	SSI	9 patients (one interviewed twice). Some (unclear how many) had carer also participating	3 female, 6 male Age range: 43–72 5 married, 3 divorced, 1 widowed 5 university educated, 2 college, 1 high school, 1 unknown 3 bulbar onset, 6 spinal Months since diagnosis: 16–132 Months since respiratory failure: 2–52 5 using NIV, 1 using LTMV, 1 using NIV and LTMV, 1 using neither		Six themes: the meaning of the intervention, the importance of context, the importance of values, the effect of fears, the need for information, adaptation to or acceptance of the intervention	33
Stavroulakis et al.^38^ UK	This study explores the experience of gastrostomy insertion from the perspective of the patients and their informal carers	Starting specific treatments	Thematic analysis	SSI	10 patients, 8 carers	See Stavroulakis et al.^36^ (above)		Three main themes: challenges of gastrostomy, benefits of gastrostomy, education on gastrostomy feeding/management	34
Gysels and Higginson^39^ UK	This paper compares the experience of breathlessness in cancer, COPD, heart failure and MND, four conditions sharing heavy symptom burdens, poor prognoses, high breathlessness rates and palliative care needs	Starting specific treatments (symptom)	Thematic analysis	SSI and participant observation	48 patients, 10 with MND	Patients 1 female, 9 male Age range: 24–77 (median 42)		Framework applied: the nature of breathlessness, label, timeline, cause, treatment/coping with breathlessness, treatment/coping with MND, consequences	33
Greenaway et al.^40^ UK	To identify factors associated with decisions made by patients with amyotrophic lateral sclerosis (ALS) to accept or decline non-invasive ventilation (NIV) and/or gastrostomy in a prospective population-based study	Decision-making/information seeking, starting specific treatments	Thematic analysis	SSI	21 patients	8 female, 13 male Age range: 41–76 Intervention: 4 NIV, 16 gastrostomy, 1 NIV and gastrostomy		Three main themes: (1) patient-centric factors (including perceptions of control, acceptance and need, and aspects of fear); (2) external factors (including roles played by healthcare professionals, family, and information provision); and (3) the concept of time (including living in the moment and the notion of ‘right thing, right time’).	33
Ando et al.^41^ UK	To qualitatively explore how people with MND experience NIV and how this changes over time as the illness progresses	Starting specific treatments		Multiple SSI over 12 months	5 patients	Gender: Male 4, Female 1 Age: Mean: 59 years, Range: 51–75 years Illness duration: Median 29 months Range: 23–237 months Onset type: bulbar 3, limb 2 Months with NIV: Mean: 13 months, Range: 12–14 months Relationship to caregiver: Spouse/partner 3, friend 1, sibling 1	IPA	Three superordinate themes emerged: experiences of NIV, influence on attitudes and perceived impact of NIV on prognosis.	33
Sundling et al.^42^ Sweden	To describe the patients with ALS and their caregivers’ experiences of non-invasive positive pressure ventilation	Starting specific treatments	Qualitative content analysis	In-depth interviews	7 patients, 8 caregivers	Patients: Age range: 45–75 years All living at home and had been on home ventilation for a period of 3–15 months using the ventilator daily for 2 h up to 20 h (median: 12) Carers: Age range: 40–74 years 8 partners 3 employed, 1 working as carer, 4 retired		Three main themes emerged: ‘Getting to know the ventilator’, ‘Embracing the ventilator’ and ‘Being on a ventilator on a 20 h-24 h basis’	32
Baxter et al.^43^ UK	To assess whether patient use of non-invasive ventilation (NIV) impacted on their family carer, and to explore other sources of carer burden	Starting specific treatments	Thematic analysis	Qualitative interviews (and scores – mixed methods)	16 family carers	Patient ages: 1 = under 60, 6 = 60–69, 9 = 70+ Onset: 1 bulbar, 2 respiratory, 13 limb 14 partner, 1 child, 1 other family member		Two headings: carer perceptions regarding the impact of NIV; carer perceptions regarding the source of burden (role change and patient needs, difficulty having time away, acceptance of professional help, timing of equipment and supportive services).	33
Baxter et al.^44^ UK	To examine the experiences of patients with motor neurone disease and their carers following the recommendation to use non-invasive ventilation (NIV)	Starting specific treatments	Thematic analysis	SSI	20 patients, 17 carers	Patients: 5 female, 15 male Mean age: 67 17 married/partnership, 1 divorced, 2 widowed 16 limb onset, 3 bulbar, 1 respiratory NIV usage: 13 regular, 3 low, 4 non-user		Perceived barriers, perseverance, perceived benefits	27
Baxter et al.^45^ UK	To describe carer and health professional experiences of end-of-life care of motor neurone disease patients using non-invasive ventilation	Starting specific treatments, end of life	Thematic analysis	SSI	9 family carers, 15 HCPs	Carers: 8 partners, 1 child (of people with MND) (Patients: 7 limb onset, 2 bulbar, 1 respiratory)		9 key themes: 1. Unexpected speed of deterioration 2. Hospitalisation versus dying at home 3. Attempts to resuscitate 4. Decision-making regarding the withdrawal of NIV 5. Peaceful final moments 6. Turning off the machine 7. Professional uncertainty regarding the use of NIV 8. Positive impacts of NIV use 9. Concerns regarding NIV use	30
Siewers et al.^46^ Norway	To explore patients’, family carers’ and health professionals’ experiences with using mechanical insufflation – exsufflation (MI-E) in amyotrophic lateral sclerosis (ALS) in the home setting	Starting specific treatments	thematic content analysis, as described by Malterud	SSI	5 patients, 3 carers, 3 HCPs	1 female, 4 male Age range: 43–81		We identified several themes related to patients and their carers’ experiences with using the MI-E device: trust and confidence, learning to use the device, individualised use of MI-E and perceived effects.	33
Akiyama et al.^47^ Japan	To explain the experiences of caregivers of patients with amyotrophic lateral sclerosis (ALS) receiving invasive ventilation in Japan	Starting specific treatments		SSI	12 primary caregivers	10 female, 2 male Mean age: 56.1 years 9 spouses, 2 parents, 1 child	Grounded theory	Caregivers tried to ‘find a meaning in prolonging life,’ which represented a core category. Two subcategories relate directly to the core category. These were ‘hesitation and regret over the decision’ and ‘feeling of being supported’. Each subcategory had four internal dimensions: ‘uncertainty of the future’, ‘communication’, ‘maintaining their own life’ and ‘support’	33
Veronese et al.^48^ Italy	To identify how the decision of a tracheostomy was taken by the patients, and collect qualitative information from family carers about the end-of-life phase of ALS patients who died after being tracheostomised and mechanically ventilated, looking in particular at the possibility of the prediction of the end of life in these patients and the possible involvement of specialist palliative care	Starting specific treatments, end of life	Content analysis (thematic)	SSI	19 family caregivers	11 spouses, 7 children, 1 paid carer who lived alone with the patient for 5 years The interviews were performed after a mean period of 2.8 years after the patients’ death (range: 6 months to 7 years) (Patients: 9 male, 10 female Mean age: 57 (range: 30–72) Mean survival from diagnosis 24 months (range: 16–156)		There were two main areas that could be seen from the interviews: the ‘process of consent’ to the tracheostomy and the ‘predictability of deterioration at the end of life’	29
Herz et al.^49^ Australia	To explore the experiences and perceptions of carers and former carers of people with MND with emphasis on the later stages of the disease	End of life	Thematic analysis	Focus groups	11 carers (3 current, 8 former)	Current carers: 1 female, 2 male One aged <35, one 36–45, one 76–85 2 partner, one child (of person with MND) two 0–6 months since diagnosis, 1 >12 months since diagnosis Former carers: 6 female, 2 male 2 aged 36–45, 2 aged 46–55, 4 aged 56–65 4 partners, 2 children, 2 de facto carers two 0–12 months since death, two 13–24 months since death, four 25–36 months		Headings: role of the general practitioner, role of the MNDA, unremitting care, emotional cost to the carer, need for respite, accessing help, love, suspension of needs, trapped and drowning, financial burden, access to palliative care, return to living	30
Rosengren et al.^50^ Sweden	To describe patients’ experiences of living with ALS in the end-of-life situations	End of life	Qualitative content analysis	Written narratives (autobiographies)	4 patients	4 female, 0 male		The categories suffering, meaningfulness and experiences of a limited life were identified as describing patients’ understanding of living with ALS.	28
Ray et al.^51^ Australia	To examine the ways, family caregivers of people living with motor neurone disease (MND) experienced the dying of their relative and to identify how health practitioners can better prepare families for end-of-life care	End of life	Supplementary analysis (secondary analysis)	Interview and observational data	18 family caregivers (Australia) 11 family caregivers (England)	Ratio male: female = 1.4:1 All carers were partners except 1 (who was child of person with MND)		Combined data revealed four major themes: planning for end of life, unexpected dying, dignity in the dying body and positive end to MND.	33
Bentley and O’Connor^52^ Australia	To examine the perceptions of end-of-life experiences of family carers of people with MND in Western Australia (WA) to identify unmet needs and gaps in end-of-life support for people with MND and their family carers	End of life	Thematic analysis	SSI	12 bereaved carers	Gender: Male 5, female 7 Relationship to deceased Spouse/partner 11 Child 1 Age 45–49 (2) 50–59 (0) 60–69 (8) 70–79 (2) Place of death: home 8, hospital 2, hospice 1, residential facility 1 Time from diagnosis to death Less than 1 year (4) 1–2 years (3) 2–3 years (4) 3–4 years (–) More than 4 years (1) Time bereaved 3–6 months (7) 7–12 months (3) 12–15 months (2)		The themes identified can be summarised into three main areas: accessing support, accessing information and feeling prepared.	35
Whitehead et al.^53^ UK	To explore the experiences of people with motor neurone disease (MND), current and bereaved carers in the final stages of the disease and bereavement period	End of life, bereavement	Thematic analysis	Narrative interviews	24 patients, 28 carers (18 current, 10 former)	See O’Brien et al.^19^ (above)		Themes: anxieties, end-of-life decision-making and advance care planning, services as the end-of-life stage, impact on carers, euthanasia	33
Solomon and Hansen^54^ USA	To explore the unique lived experiences of one patient who died at home and her family members, and to interpret how dying at home influenced patterns of bereavement for this patient’s family	End of life, bereavement	(Thematic analysis)	In-depth telephone interviews	1 patient, her husband and 3 children	Patient: 78-year-old white female, diagnosed 6 months prior to death Husband 79 years old Children 52–58 years old	Benner’s interpretative phenomenological approach	Paradigm case: the meaning of being at home (exemplars – driving her own course, not being a burden). Themes contributing to a successful death: patient characteristics, family characteristics, support, emotions, time, aspects of the healthcare team	32
Aoun et al.^55^ Australia	The aim of this study was to explore the experiences of MND family carers from their time as a carer of their spouse through to their bereavement	Bereavement	Thematic analysis	Semi-structured interviews	16 bereaved family carers	13 female, 3 male All partners of MND patients Age range: 53–81 (mean: 65) Beareaved between 1–4 years (mean: 27.5 months) *Patient age range: 50–82 (mean: 65)* *Length of time to obtain initial diagnosis 1–12* *months (mean: 5.8* *months)* *Time from diagnosis to death 3* months to *6* years (*mean: 22* *months*) *All 16 offered palliative care*		Five themes – the work of family carers, the change in relationship from spouse to family carer, family caring as a series of losses, coping mechanisms of family carers and supportive and palliative care experiences of family carers. The six participants who met the criteria for prolonged grief disorder accessed palliative care at a later stage in the disease trajectory.	34
Penrod et al.^56^ USA	To illustrate variations in caregiving trajectories as described by informal family caregivers providing end-of-life care	Trajectories		Unstructured interviews	46 caregivers (10% ALS) 1 ALS case study	ALS case study: 38-year-old female caring for husband	Grounded theory	The unifying theme of end-of-life caregiving is ‘seeking normal’ as family caregivers worked towards achieving a steady state, or sense of normal during their caregiving experiences.	29
Shipley^57^ USA	Aim 1 was to document the life patterns of family caregivers of ALS patients exhibited through the nurse researcher/ALS family caregiver process of health as expanding consciousness (HEC). Aim 2 was to integrate the life patterns of individual family caregivers of ALS patients into a thematic pattern of the whole representing the ALS caregiving experience across all caregiving families	Trajectories		SSI	8 family caregivers	4 female, 4 male Age range: 27–85 4 working full time, 4 retired 7 partners, 1 child 8 white ethnicity	Hermeneutic dialectics	Nine patterns: (1) suspicions emerge but ALS diagnosis is delayed, (2) support that helps the caregiver, (3) support can make caregiving more difficult, (4) looking towards the future, (5) adaptations from ALS, (6) obstacles to the caregiving role, (7) caregiver respite, (8) focus of others and (9) strategies aiding the caregiving role. The nurse researcher/ALS family caregiver process that was revealed in this research study was (1) establishing a time and place for the nurse researcher and ALS caregiver to form a relationship, (2) developing a bond with each ALS caregiver, (3) creating an atmosphere which allows the caregiver and nurse complete freedom to express themselves openly, (4) offering a sense of timelessness for insights about the ALS caregiving experience and (5) transformation as the nurse researcher and ALS family caregiver came together to find meaning in the chaotic experience of family caregiving for an ALS patient.	35
Harris et al.^8^ England	To explore the meaning of living with uncertainty for people diagnosed with MND	Throughout illness trajectory	Hermeneutic (interpretive) phenomenological project	Semi-structured interview	Four people with MND	Recruited from the MNDA Care Centre in northwest England; inclusion criteria were accepted diagnosis of MND (all types), above age 18, had been receiving care and treatment for 3–6 months. Individuals were excluded if they were deemed unable to provide informed consent	Interpretive description	Three aspects of the illness trajectory of MND: (1) body failing prematurely and searching for answers, (2) body deterioration and responses to care, (3) body nearing its end and needing to talk	33

SD: standard deviation; MND: motor neurone disease; IQR: interquartile range; HCPs: healthcare professionals; COPD: chronic obstructive pulmonary disease; MNDA: Motor Neurone Disease Association ; SSI: semi-structured interview; PBP: progressive bulbar palsy; PMA: progressive muscular atrophy; PLS: primary lateral sclerosis; LTMV: long-term mechanical ventilation; MI-E: mechanical in-exsufflation.

### Conducting the synthesis

The review was conducted using thematic synthesis,^13^ an established methodology for synthesising the findings of multiple qualitative studies that enables the development of both descriptive and analytical themes from the body of included papers.^58^ Following the stages of thematic synthesis, the text was processed line-by-line and individual codes were identified and checked for consistency. Coding was carried out by one reviewer (V.T.) and checked by a second for consistency (K.F.). In order to determine individual’s experiences across the disease process, papers were broadly coded as to their focus within the disease trajectory; for example, those papers detailing experiences of diagnosis were coded prior to papers exploring issues of deterioration, end-of-life care and bereavement. Once each article had been coded, the individual codes were organised into broader groups to develop descriptive themes. These themes were then synthesised further to produce the analytical themes. Each of these discrete stages were determined through discussion with the project advisory group, which included an expert-by-experience and palliative care physician, and were felt to reflect key stages experienced in the life-course of people with motor neurone disease and carers. As a result, the derivation of themes was predominantly inductive, although there was a deductive component as the process was framed by consideration of disease trajectory detailed above. Atlas.ti software was used to manage this process.

## Results

### Results of searching, inclusion and quality appraisal

**Figure 1.**
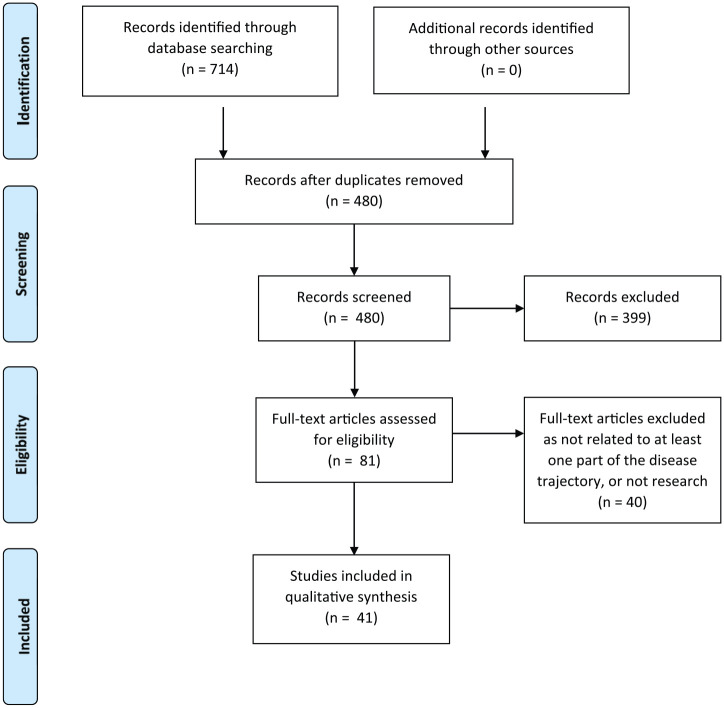
PRISMA flowchart of included/excluded studies.

The searches of electronic databases identified 480 unique results, of which 399 were excluded following title and abstract review, leaving 81 studies to be assessed for full eligibility (Figure 1). Mapping the papers against the stages of the disease trajectory led to the inclusion of 41 papers (Table 2) whose aims were relevant to one or more disease trajectory stages. Papers that were excluded were revisited at the end of the review process to identify any additional themes that had been missed by excluding the generic literature.

Study quality appraisal scores were generally good:^16^ seven papers scored between 25 and 29, and 33 scored 30 or greater (out of 36).

### Characteristics of the included studies

The majority of the included studies were published in the United Kingdom (19), followed by Australia (6), Sweden (4), Ireland (3) and the United States (3). There was one paper each from Canada, Germany, Italy, Japan, the Netherlands and Norway.

The studies’ foci were carers (*n* = 13), patients (*n* = 12), both carers and patients (*n* = 2), carers, patients and healthcare practitioners (*n* = 2), carers and healthcare practitioners (*n* = 1) and patients and healthcare practitioners (*n* = 1). Findings from healthcare practitioners were excluded from this review as the focus populations were patients and carers.

A total of 40 papers were published peer-reviewed articles and 1 was a PhD thesis. A number of studies produced multiple publications;^19,24,27–29,34–36,38,53^ therefore, 33 studies were included, reported across the 41 papers.

### Characteristics of the included participants

The included papers represented experiences of 358 people with motor neurone disease and 369 carers. The majority of papers reported demographics of the participants (see Table 2). Of the participants with motor neurone disease: the age range was 21–85 years; 45% were female; reported time to diagnosis was 3–60 months; and the overall time since symptom onset was 0.6–237 months. Types of motor neurone disease were reported under variable headings, with 48 given as ‘limb onset’, 36 as ‘bulbar onset’, 32 as ‘spinal onset’, 3 as ‘respiratory onset’, 8 as ‘amyotrophic lateral sclerosis’, 5 as ‘progressive bulbar palsy’, 3 as ‘primary lateral sclerosis’ and 2 as ‘progressive muscular atrophy’. These characteristics broadly represent the epidemiology of motor neurone disease.^59^

Where demographic data were reported for carers, the age range was 25–86 years, 65% were female, 78% were partners of someone with motor neurone disease, 16% were adult children, 6% were other carers (e.g. parent). At time of interview, carers had been bereaved between 2 months and 7 years or had been a carer between 6 and 8 years.

### Results of the methods of thematic synthesis

**Figure 2.**
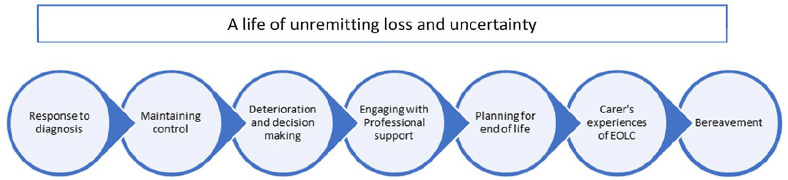
Disease trajectory points.

The results of line-by-line coding led to the development of 361 codes. These were then analysed by the reviewers for similarities and grouped into 10 descriptive themes (Table 3). These descriptive themes detailed patients’ and carers’ experiences of living with motor neurone disease, capturing provision of and need for palliative and end-of-life care where it was described. In order to ‘go beyond’ the content of the original papers^17^ and develop the thematic synthesis, the descriptive themes were synthesised further against consideration of the disease trajectory. As a result, a series of seven analytical themes were developed that represent patients’ and carers’ experiences of living with motor neurone disease, across the course of its trajectory, overlaid with an overarching theme running through the trajectory of unremitting loss and uncertainty (Figure 2).

**Table 3.** Descriptive and analytical themes.

Descriptive themes	Analytical themes
Effect on patient/carer Attitude to dying Attitude to future Communication Decision-making Healthcare provision Support Information Caring Bereavement	Response to diagnosis Maintaining control Deterioration and decision-making A life of unremitting loss Engaging with professional support Planning for end of life Carers’ experiences of end-of-life care Bereavement

## Findings

The findings of the review are presented against each of the seven analytical themes identified and detail patient and carer experiences of, and need for, palliative care, spanning the illness trajectory associated with motor neurone disease (Figure 2).

### Response to diagnosis

Due to delays of up to 60 months between first symptoms and diagnosis with motor neurone disease, for many individuals, the diagnosis came as a shock; for others, it confirmed their expectations. Many negative reactions prevailed, including bewilderment,^19,20,23,27^ denial^18–20,23,35,51,57^ and a sense of loss and sadness.^8,23,37,57^ At the extremes were people who became very distressed by their position and those who experienced suicidal thoughts:^19,20,23,37,39^

. . . you don’t understand the news, you deny it, become very despairing. (Patient)^23^ (p. 502)

I was expecting it, it wasn’t a surprise. I was pretty much even convinced before I went to see him that this is what I had . . . Although you expect it and it is not a surprise, it is still a bit of a shock. (Patient)^35^ (p. 832)

Individuals and carers stated a clear need for follow-up support immediately after the diagnosis, recalling it as a very vulnerable time with no indication of impact, what diagnosis might mean in reality or what the future would hold:

There was no nurse support, no quiet room to reflect and no support material. The lack of support that day was profound. (Patient)^19^ (p. 101)

. . . and nothing, absolutely nothing was given to him, nothing at all. Not ‘do you want us to contact your wife, do you want some sedation for the weekend?’ He was just left in pieces. (Former carer)^19^ (p. 101)

### Maintaining control

In response to diagnosis, people sought to maintain control of their lives, keeping a sense of normality and purpose where possible. Some questioned what had caused the disease, although recognising there may be no clear cause:

I am trying to be brave for the likes of my wife’s sake and the children, you know. (Patient)^20^ (p. 162)

Because I’m such a healthy person, and to get something like this, you think ‘OK, what did I do?’ It’s not right. (Patient)^35^ (p. 832)

Ways of maintaining control included managing stress and protecting time for themselves, both as individuals and together.^21,47,49,57^ Participants had varying attitudes to how they approached inevitable deterioration, with some wishing to plan ahead^8,20,22,28,29,34,35,39,40,48,54,56,57^ and others preferring to live in the present and not consider the future.^22–25,28,31,33–35,39,40,50,51,56,57^

As the disease progressed, both patients and carers tried to find ways to counteract feelings of loss and uncertainty, and to find new meaning in their life:^21–23,25,29,35,37,47,49,50,56,57^

Determination to do what I can for as long as I can, desire for independence [and a] resilient temperament. (Patient)^35^ (p. 834)

### Deterioration and decision-making

Decision-making during became particularly important during periods of deterioration with a focus on interventions and advance care planning. Decision-making processes were complex and multifaceted and supported individualised (rather than ‘algorithm-based’) approaches.^40^

Physical difficulties experienced during periods of significant deterioration included changes in speech/communication,^18,23,25,27,29,32,34,37,42,43,47,48,50,51,53,56,60^ breathing^24,29,32,37,39,41,42,46^ and eating/swallowing,^25,29,36,40,51,53,57^ alongside general decline in mobility from increasing muscle weakness.^24,27,29,34,37,53,56,57^ Breathlessness and communication difficulties caused most distress. As deterioration progressed, decision-making in the context of interventions to mitigate against the loss of function became central, for example, the introduction of percutaneous endoscopic gastrostomy feeding tubes and non-invasive ventilation.^21,24,27,29,34–36,39,48,53,57^ Such interventions were seen as ‘life-sustaining’ treatment; they allowed patients to maintain a degree of function they could not otherwise attain. Much of the discussion on aids/equipment concerned when to start using them and the impact they had on patient and carers’ lives, although delays in initiation often occurred.^34,37,40,57^

Typically, decisions to commence a physical intervention followed a crisis situation, such as acute worsening of respiratory distress.^36,37,44,48^ However, timing of decisions was also driven by personal choice. While carers and healthcare practitioners played an important role in decision-making, the majority of patients preferred to have the final say:^24,32,34–37,40,42,44,45,51,53,54,57^

In the beginning it [MI-E (mechanical insufflation – exsufflation)] was just placed there (smiles). I had all different kinds of excuses why I wouldn’t use it. But now I’ve realized that it helps me. (Patient)^46^ (p. 204)

Consideration of physical interventions to palliate symptoms of deterioration led to ‘life or death’ scenarios for individuals; without intervention, they were likely to die, and with it, they would live for longer.^24,25,29,35,40^ While a few people wished to live for as long as possible with high levels of disability, the majority felt life-sustaining treatment only prolonged suffering, prompting debate around maintaining quality versus quantity of life:^22,24,25,29,41^

Dragging it out. Well I don’t see the point . . . I think people might incorrectly believe that a life-sustaining treatment might lead to a general improvement . . . the feeling of having something [like this] dragging on and on. I don’t want that. (Patient)^29^ (p. 321)

Generic prognosis advice was seen as misleading, as there was considerable variation in duration of illness and onset of particular symptoms.^19,35,40,47,51,52^

Information gathering was important to help inform and guide decision-making. Significant variation existed between individuals as to the degree of information required, its timing and sources.^19,31,33,35,37,40^ Guidance from health professionals was valued in supporting individual’s information needs, although commonly information was seen as insufficient or too generalised.^19,26,33,37,38,40,47,48,50,52,55,53,57^ Obtaining the balance between the right information at the right time was delicate and rarely achieved:

If you get too much information, you start worrying, waiting for it to happen . . . I don’t want information . . . it would make me depressed. (Patient)^31^ (p. 967)

Carers became an important source of information, acting as ‘experts’ for both patients and healthcare practitioners.^30,34,42,49^ They provided patient-specific information for healthcare practitioners and disease-specific information for both patients and healthcare practitioners.^20,31,34,35,38^

### Engaging with professional support

Multi-disciplinary support was useful during periods of deterioration and associated decision-making. Co-ordination and continuity of care were seen as key, as was involvement and support in decision-making. Individuals appreciated the specialist knowledge and understanding they got from professionals in the multi-disciplinary team and having a single point of access into the health system.^19,27,28,34,35,39,40,49,52,56,57^ Case managers were particularly valued: having more time than other multi-disciplinary team members and being proactive in tackling problems.^28,33^ In addition, they were seen to relieve some burden from family carers:^27,39,53,56^

The clinic here is very good. The thing that I liked about it is that you see all the different disciplines on the one day, so it’s not five different visits to the hospital . . . it takes about 2 to 3 hours to get him out of the house. So this is much easier’. (Carer)^34^ (p. 177)

The volume of different people involved in multi-disciplinary team care could, however, be overwhelming^8,20,24,26,27,45^ and exacerbated by people in non-specialist support roles who were unsure how to manage people with motor neurone disease.

### Planning for end of life

Individuals’ preferences in relation to the end of their life were highly personalised, although commonalities emerged regarding perceptions of a good death. These included an absence of suffering, a quick and pain-free death, dying with dignity, having family present and maintaining control up to and including the end of life.^23,24,29,32,35,40,41,44,47,49–51,53,54^ A preference to die at home was expressed by most individuals, with hospital death being viewed negatively:

I really do not want to die in hospital unless it is absolutely necessary. (Patient)^53^ (p. 373)

The finest, softest, most wonderful event that could happen to me would be to die with dignity. Get to sleep quietly one evening. I have said goodbye to all the friends. (Patient)^50^ (p. 78)

Assisted suicide and euthanasia were talked about by both patients and carers in a small number of studies. In all cases, these views were expressed in countries where any form of assisted dying was illegal, or data were collected prior to a change in law.^23,24,37,50,53^ Considerations of assisted death were connected with a wish to die before the ‘final stage’ of the illness in order to maintain a sense of control. Of particular concern to people was experiencing further physical deterioration, of having an active brain stuck in a dysfunctional body and being a burden to others as a result. A quick death, ideally from another cause, was seen as the ideal answer to this by some.^23,24,50,53^ For a few, assisted suicide or euthanasia were perceived as options, if available, that would help them live more easily alongside the knowledge of their deterioration:^23^

I need to be able to communicate and if I can’t communicate then I’m not quite sure about whether I’d rather or not have some sort of euthanasia by then, I don’t know. (Patient)^53^ (p. 375)

I would be much calmer if euthanasia were permitted. It would make it much easier for me to live. I do not want euthanasia now . . . If I knew I could get help dying, my quality of life would be higher. It disturbs me that I cannot have this help.^23^ (p. 502)

Those who were bereaved carers acknowledged that there had been limited discussion about plans for the end of life:

I think we were in denial for quite some while you know, we knew it was coming, but we didn’t plan anything about it. (Carer)^51^ (p. 469)

### Coping with caring

An individual’s deterioration affected their carers by increasing the need for physical support: working ventilators, preparing feeds and conducting personal care around the clock. Such work created a significant burden physically and mentally, which could lead to ill health and confinement at home.^34,36,45,48–51,53,56,57^ Carers also negotiated logistical challenges through coordinating care and daily activities.^38^ Through this activity, carers experienced poor sleep, both due to nightly care needs and noise from ventilators and often found themselves exhausted from the effort of constant care:^49^

I was working like a zombie because I would be nursing him all night [and working all day]. (Carer)^49^ (p. 212)

Caring affected working and social lives, presenting difficulties with maintaining employment and limited opportunities to see family and friends or have personal time. Carers were concerned about their ability to cope with increasing care needs and, after death, had fears about being left alone and living without purpose. Caring had a significant emotional impact, both positive and negative. Positive emotions included love, admiration, resilience and maintaining a positive outlook,^22,54,56,57^ contrasting with negative emotions of anxiety, anger, fear, sorrow, despair and loneliness.^22,26,45,47,49,52,55–57^ Carers endured high levels of stress, often feeling overwhelmed by the constant situation they faced:

I always felt this pressure just inside like holding everything in and occasionally little spurts would come out whether it be crying or just you know losing my patience with the kids or other things . . .’ (Carer)^56^ (p. 5)

Despite this, carers were often unwilling to share the burden of caring with others, considering they held ultimate responsibility and were best equipped with in-depth knowledge of the patient’s needs. Carers expressed concern when leaving patients with other people:

I knew I could nurse him better at home, 24 hours, than what I could nurse him [in the hospital]. I was in there 11–12 hours a day, and half the night. (Former carer)^49^ (p. 211)

Carers generally put their own needs last, hiding their feelings and being unwilling to share the caring burden with anyone else.^22,26,34,45,47,49,55,57^ They became resilient in the face of adversity, exceeding their own expectations.

While carers shouldered most of the patient’s day-to-day burdens, external support was provided by friends and family. Support also came from religious communities, other people with motor neurone disease and their carers.^21,25,28,29,34,39,47,50,53,57^ Motor neurone disease support groups (both online and face-to-face) provided practical advice and helped people normalise their position;^25,33,47,49,57^ however, some felt uncomfortable being in the company of people with more advanced disease or bereaved carers.

### Carers’ experiences of end-of-life care

Where decision-making and advance care planning had occurred, there were reports that these wishes were not adhered to at the end of life, leading to incidents where care requests were ignored:^51–53^

When I went in the ambulance to (local hospital) I took this [Preferred Priorities of Care document] with me because that’s what Specialist nurse had told me to do, take that with you [. . .] so when I see the A and E doctor . . . and I showed him the part where it says in the event of serious collapse, the patient does not want to resuscitated, but the A and E doctor said ‘well it’s not worth the paper it’s written on, what are you talking about?’ (Former carer)^53^ (p. 372)

Occasionally, carers were taken by surprise by the end-of-life phase, with individuals’ deaths occurring unexpectedly, leading to additional distress.^44,51,52^ In part, this was perceived to be a result of inadequate communication by healthcare practitioners identifying that individuals were in the terminal phase.^52,53^ Although not all deaths could be predicted, certain key features were mentioned by carers that, with hindsight, indicated the end of life was approaching. These features included particular symptoms, such as loss of communication and clinical complications secondary to advanced disease progression.

During the last days of life, there was a perceived lack of external support for individuals and carers, with poor access to services. Across the papers, the last days of life were commonly the first time individuals and carers had discussed specialist palliative care support,^29,49,51–53,55^ with the exception of one paper.^8^ Carers expressed serious concerns regarding lack of knowledge around end-of-life care among healthcare professionals in acute and community services which, in turn, impacted on healthcare practitioners’ willingness to engage with palliative care services and seek profession-to-professional support. Carers also felt that even when such services were involved, inexperience or lack of knowledge for the needs of people with motor neurone disease existed,^8^ with carers struggling to find answers about the dying process:

The last 3 weeks of my mother’s life . . . and they made it terrible absolutely terrible, well it was horrific . . . through their incompetence and through their not knowing, the lack of knowledge of motor neurone . . . (Former carer)^30^ (p. 506)

Delays in accessing care and the inexperience of community staff dealing with motor neurone disease meant that not all the required care was received, undermining an individual’s wishes to die at home:

Ideally, if somebody had said, look, we know it’s getting towards the end, and somebody will move in with you for part of the day or something because it was imminent. It would have been so much kinder for him to die in his own surroundings. (Former carer)^53^ (p. 373)

### Bereavement

Where papers included the experiences of bereaved carers, people talked of both positive and negative outcomes from both their caring experiences and the process of death.

Carers’ bereavement experiences were affected by the manner in which the person they cared for died. Perceived good deaths generally aided a more straightforward bereavement, whereas traumatic or sudden/unexpected deaths made the bereavement process more difficult.^51,54–56^ For those who only gained access to palliative care later in the disease trajectory, or who were unwilling to accept/plan for the future, tended to experience more prolonged grief during bereavement.^55^ On reflection, some carers were able to focus on the positive care they had provided, but some felt guilt and regret^19,51,53,55^about things they felt they could have done better. It was acknowledged that bereavement was a slow process:^44,49,51,53^

But . . . I can’t stop thinking about him . . . being on the floor. But you don’t think about what you did. It’s the one thing you didn’t do [that you think about]. It was awful. (Former carer)^53^ (p. 374)

Carers described a lack of support around bereavement,^53,55^ and that this came as a shock given the extensive input they had had up until the person with motor neurone disease died:

When (patient) died (Specialist nurse) never got in touch with me . . . I was absolutely devastated about that, I couldn’t get over it, couldn’t get over it . . . You are just cut off . . .’ (Former carer)^53^ (p. 374)

### A life of unremitting loss and uncertainty

Overlaying the disease trajectory was the enduring experience for both patients and carers of a life of unremitting loss and uncertainty. Deterioration and its physical, emotional and social sequelae had a significant effect on the lives of both patients and carers.^8,18–57^

The feelings of loss spanned the disease trajectory, with the cumulative losses (physical, emotional, social) most pronounced during the phase of acute deterioration:

What was important to me was always the family, then food, drink and conversation. I can’t do anything of it now. Huge, unbelievable loss. (Patient)^24^ (p. 115)

There is so little time to take it on board, it’s [loss] incessant. The future is so grim because of all the loss. (Patient)^24^ (p. 115)

Loss was experienced in parallel with great uncertainty over the future, both immediate and longer term:

You don’t know how it’s going to proceed . . . whether it’s going to proceed quickly . . . the uncertainty makes it far more difficult to make decisions because you don’t know what tomorrow’s going to bring. I mean I’m sure [patient] would find it easier, if she knew how it was going to progress and if we had some idea of timescale. (Carer)^36^ (p. 60)

It’s the not knowing, that’s the most frightening bit, if they could say right you’ve got two years that’s it, I’d think well that’s fair enough, I can work towards that but it’s the not knowing. (Patient)^53^ (p. 371)

Most concerning for people was the fear of not knowing what was coming next. This was closely associated with the speed with which the disease progressed and the difficulty of keeping up with the present. Lack of control over the disease and its progress underpinned this uncertainty:

I’m not afraid of dying, I’m just afraid of how I’m going to die.^53^ (p. 371)

## Discussion

### Main findings of the study

This review reports on the synthesised experiences of 358 individuals with motor neurone disease and 369 informal carers; it explores living with motor neurone disease and identifies exposure to, and experience of, palliative care across its trajectory. A key finding of the review was that individuals’ and carers’ experiences of palliative care were very limited, with no explicit discussion until the ‘end-of-life’ stage of the illness; this appeared to be the only point any exposure to palliative care occurred.

Diagnosis was recalled as a very vulnerable time by both patients and carers where they would have valued additional support. Reframing palliative care to the start of the disease trajectory, ensuring prompt access and encouraging health professionals to initiate advance care planning early the disease, could become part of routine specialist motor neurone disease care.^61^ While early referral to palliative care was discussed in related literature nearly two decades ago, it infrequently occurs.^62^ Within the review, people who had been supported to plan ahead and were able to access palliative care services earlier had a less traumatic disease experience and experienced less prolonged grief in the bereavement phase. Alongside this, however, sat experiences in which advance care planning had occurred, with clear ceilings of care identified, which were not adhered to when an acute event occurred.

Individuals strove to maintain autonomy in their decision-making particularly at points of deterioration; however, deterioration was not generally supported with an increase in care and support. Throughout the review, decisions around treatment interventions to palliate symptoms were generally the closest that individuals got to talking about palliative care.

Early and effective communication, ideally with a single point of contact/key worker and integrated within multi-disciplinary teams with experience of caring for people with motor neurone disease, can help individuals plan for deterioration and individual ceilings of care accordingly.^61^ Anticipating and discussing intervention options to manage possible trajectories of deterioration is key, in combination with advance care planning. Palliative care can provide a platform for this to occur through the use of advanced communication skills and support for decision-making.^63^ Access to such support would enable patients and carers to have a better overall experience of living and dying with motor neurone disease and help mitigate against the inevitable loss and uncertainty felt as a result of the disease.^64^

It is important to consider that individual narratives within the review demonstrated a range of different preferences regarding the information, interventions and services, with varying attitudes to, decision-making, the future and ultimately death. Any framework for improving palliative care for people with motor neurone disease and their carers (or palliative care more widely) should take account of individual variability and the need for autonomy.^65^ In relation to individual’s experiences through the disease trajectory, preparation for each phase should ideally take place within the previous phase. Recent work identifying physical triggers for palliative care for those with progressive neurodegenerative conditions could help support this integration.^60^ It should be noted, however, that the disease trajectory may not be a smooth continuum with stages being missed or jumped around in an individual’s journey.

The review demonstrated that carers are affected by motor neurone disease in many similar ways to those living with it. They have similar emotions, are subject to the same confinement and isolation through their caring role, struggle to balance loss and maintaining control. Caregiver burden tends to increase as individual’s behavioural and physical impairments worsen, with carers carrying the additional burden of anticipatory grief.^66^ Lack of specialist knowledge of motor neurone disease in community teams, particularly the acknowledgement that death was imminent, further added to this burden. Such lack of knowledge is an area identified for development from the review and is reflected elsewhere in the literature.^63,65^ Carers additionally recognised the potential need for support after death and talked of how lack of palliative care or palliative care occurring only late in the disease process impacted on their bereavement.

Individuals’ and carers’ described their experiences of motor neurone disease as a cycle of unremitting loss and uncertainty, with individuals having a desire to maintain control of their lives. Key to ensuring a sense of control is enabling individuals to maintain their autonomy.^65^ Support for patients and carers, both individually and as a pair, is vital to helping people adjust to such loss and maintain a sense of control.^67^ The holistic and broad nature of support available through palliative care services can provide a path through loss, anticipatory grief and into bereavement.^68^

### Strengths

The review includes an extensive international qualitative literature detailing individuals’ experiences. Findings are therefore directly drawn from and reflect these experiences, giving power to the patient and carer voice. In order to ensure that the results from this review remained clinically relevant to health professionals and to people with motor neurone disease and their carers, input was received from a palliative care clinician and an expert-by-experience at key points in the development of the study.

The disease trajectory identified in this review reflects the early work of Glaser and Strauss.^69^ It is unique in comparison with other trajectories of palliative care for people with motor neurone disease, in that it represents the synthesised illness experiences of individuals and their carers and integrates these with key points of disease progression.^64,70^

### Limitations

The number of studies included in the review was ‘actively managed’ through purposive sampling.^71,72^ Such sampling may have excluded papers with potential to contribute to the analytic themes arising from the review. To mitigate against this, all excluded papers were re-read after the development of the descriptive and analytic themes in order to ensure there were no concepts within these papers that could have contributed to the review.

Articles for inclusion were not excluded by country of origin. While this broadens the findings to a more international focus, the papers are therefore also reflective of a disparate set of healthcare systems.

The majority of people with motor neurone disease included in the reviewed papers had a family carer and lived in their own home. People living in alternative settings had a limited voice; more research is required to understand how their needs might be different from those with full-time carers.

### What this study adds

The review highlights key recommendations (Table 4) for each part of the disease trajectory which, if implemented, could greatly improve patient and carer experiences of motor neurone disease. Many of these needs could be addressed by incorporating palliative care services or approaches into care delivery. As the recommendations arise from the synthesis of an international body of literature, they are relevant to an international audience.

**Table 4.** Recommendations arising from the synthesis findings.

Reframing palliative care to the point of diagnosis and ensuring prompt access
Patients and carers need support immediately after receiving the diagnosis, including timely signposting to key sources of information relevant to the individual patient, with repeat signposting through the disease trajectory
Single point of access or a ‘key worker’ to the multi-disciplinary team and focus for all care
Preparation for the next stage of the disease trajectory should take place during the phase before, particularly in relation to the introduction of interventions
Health professionals require support and education to initiate advance care planning early in the disease and to provide support around decision-making at points of deterioration
Any framework for palliative care should incorporate individual variability
Targeted support for carers to relieve excessive strain and fatigue amongst carers of people with MND
Education provision is required to address the lack of knowledge among healthcare professionals in acute and community services of the needs of people with MND. This needs to be balanced against the challenge of the lack of exposure to caring for people with the condition

MND: motor neurone disease.

## Conclusion

The review identified a considerable literature exploring care experiences and needs of people with motor neurone disease and their carers; however, palliative care was commonly restricted to the last few days of life. By examining commonalties of patient and carer experience across the disease trajectory, key points were identified where early integration of palliative care would allow individuals to cope better with the cycle of loss associated with motor neurone disease and maintain greater control of their own lives. It could also enable the provision of more individually tailored care, with more empowered decision-making and improved management of physical, psychological, social and other disease sequelae for patients and carers. Overall, people with motor neurone disease and the healthcare professionals caring for them would benefit from greater awareness of, and involvement with, palliative care services.
